# Recombination in Hepatitis C Virus: Identification of Four Novel Naturally Occurring Inter-Subtype Recombinants

**DOI:** 10.1371/journal.pone.0041997

**Published:** 2012-07-24

**Authors:** Weifeng Shi, Ines T. Freitas, Chaodong Zhu, Wei Zheng, William W. Hall, Desmond G. Higgins

**Affiliations:** 1 The Conway Institute of Biomolecular and Biomedical Research, University College Dublin, Dublin, Ireland; 2 Key Laboratory of Zoological Systematics and Evolution, Institute of Zoology, Chinese Academy of Sciences, Beijing, China; 3 Shenzhen Institute of Advanced Technology, Chinese Academy of Sciences, Shenzhen, China; 4 Graduate University of Chinese Academy of Sciences, Beijing, China; 5 National Virus Reference Laboratory, University College Dublin, Dublin, Ireland; Saint Louis University, United States of America

## Abstract

Recombination in Hepatitis C virus (HCV) is considered to be rare. In this study, we performed a phylogenetic analysis of 1278 full-length HCV genome sequences to identify potential recombination events. Nine inter-genotype recombinants were identified, all of which have been previously reported. This confirms the rarity of inter-genotype HCV recombinants. The analysis also identified five inter-subtype recombinants, four of which are documented for the first time (EU246930, EU246931, EU246932, and EU246937). Specifically, the latter represent four different novel recombination types (6a/6o, 6e/6o, 6e/6h, and 6n/6o), and this was well supported by seven independent methods embedded in RDP. The breakpoints of the four novel HCV recombinants are located within the NS5B coding region and were different from all previously reported breakpoints. While the locations of the breakpoints identified by RDP were not identical, they are very close. Our study suggests that while recombination in HCV is rare, this warrants further investigation.

## Introduction

Hepatitis C Virus (HCV) belongs to the family *Flaviviridae* and was first identified in 1989 [1]. It is a major cause of the liver diseases: chronic hepatitis, cirrhosis, and hepatocellular carcinoma. HCV is an enveloped virus with a positive-sense, single-stranded RNA genome of approximately 9400 bp in length [2]. The HCV genome has one open reading frame encoding a polyprotein of about 3,000 amino acids, and this is processed to produce three structural (core, E1, E2) and seven non-structural proteins (p7, NS2, NS3, NS4A, NS4B, NS5A, NS5B) [3].

Similar to many RNA viruses, HCV exhibits high genetic heterogeneity and to date seven genotypes have been identified. Different genotypes diverge by at least 30% over the complete genome [4]. In addition, HCV has also been further classified into numerous subtypes (http://hcv.lanl.gov/content/sequence/HCV/classification/genotable.html). Subtypes can diverge by as much as 20%, but within subtype variation is usually less than 10% [4]. To date, genotype 1 includes 13 subtypes (subtypes 1a to 1m). The numbers of subtypes for genotypes 2, 3, and 4 were 18, 11, and 18, respectively. Genotypes 5 and 7 have only a single subtype, 5a and 7a. However, it is likely that more subtypes might be found for these genotypes due to continuous efforts to sequence more viral genomes. Genotype 6 has the largest number of reported subtypes with a total of 21.

Recombination is an important evolutionary process for many viruses, such as human immunodeficiency virus [5] and hepatitis B virus (HBV) [6], [7]. However, recombination is considered to be rare in HCV [8], [9]. This is supported by the finding that HCV-infected cells can rarely be superinfected by another HCV of a different group or subtype, *in vivo* [10]. However, HCV super-infection or co-infection is known to occur [11]–[15] and recombination, while rare, would be expected to occur.

Recently, Gonzalez-Candelas et al. classified HCV recombination events into three types: inter-genotype recombination, inter-subtype recombination, and intra-patient/intra-subtype recombination [9]. So far, seven inter-genotype recombination types (2k/1b, 2i/6p, 2b/1b, 2/5, 2b/6w, 3a/1b and 2a/1a) and three inter-subtype recombination types (1b/1a, 1a/1c and 4a/4d) have been described, based on analysis of either full-length or partial genome sequences [9]. Specifically, the 2k/1b recombinants have been demonstrated in Russia [16], Georgia, Estonia [17], Ireland [18], Uzbekistan [19], and Cyprus [20], and these are still circulating within Europe [21], [22]. While it remains to be established, Morel et al. have suggested that genetic recombination may have important implications for HCV diagnosis, therapy, and epidemiology [21].

To date, only a few recombinants have been identified by analysis of a large number of complete genome sequences, and many recombination events have been identified by analyses of partial genome sequences [9]. It might be expected that, analysis of partial genome sequences could underestimate both the true level of recombination in HCV and may not provide an accurate identification of the breakpoints involved [9], [21]. In the present study, we have carried out an analysis of HCV recombination using the large number (n = 1278) of all available full length detailed genome sequences.

### Datasets and Methods

1278 nucleotide sequences of HCV were downloaded from the Los Alamos HCV database (http://hcv.lanl.gov/content/index) on October 5^th^, 2011. The full length HCV genome is approximately 9600 bp in size. However, only the coding regions (approximately 9000 bp) are used in our analysis. In addition, a virus sequence of canine origin [23] was downloaded from GenBank and included as an outgroup in the phylogenetic analyses.

**Table 1:** Phylogenetic evidence of four novel inter-subtype recombination events.

GenBank No.	Country	Subtype	Phylogenetic evidence of recombination			
			Fragment length: 600 bp; number of fragments: 15	Fragment length: 500 bp; number of fragments: 18	Fragment length: 400 bp; number of fragments: 23	Fragment length: 300 bp; number of fragments: 31
EU246930	Viet Nam	6a	Fragment 1–14: 6a; fragment 15: 6o	Fragment 1–17: 6a; fragment 18: 6o	Fragment 1–21: 6a; fragment 22–23: 6o	Fragment 1–28: 6a; fragment 29: 6; fragment 30–31: 6o
EU246931	Viet Nam	6e	Fragment 1–14: 6e; fragment 15: 6h	Fragment 1–2: 6e; fragment 18: 6h	Fragment 1–21: 6e; fragment 22–23: 6h	Fragment 1–28: 6e; fragment 29: 6; fragment 30–31: 6h
EU246932	Viet Nam	6e	Fragment 1–14: 6e; fragment 15: 6o	Fragment 1–17: 6e; fragment 18: 6o	Fragment 1–21: 6e; fragment 22–23: 6o	Fragment 1–28: 6e; fragment 29: 6; fragment 30–31: 6o
EU246937	Thailand	6n	Fragment 1–14: 6n; fragment 15: 6o	Fragment 1–17: 6n; fragment 18: 6o	Fragment 1–21: 6n; fragment 22–23: 6o	Fragment 1–28: 6n; fragment 29: 6; fragment 30–31: 6o

The DNA sequences were initially translated into protein sequences. The protein sequences were aligned using Clustal Omega [24] and the alignment was adjusted manually in Bioedit [25]. The DNA sequence alignment was then made using the protein alignment as a template. The DNA alignment was 9270 bp in length. We applied four strategies to subdivide the alignment into sub-datasets (Table S1). The first strategy subdivides the full-length genome alignment into 15 sub-datasets, with the first 14 sub-datasets 600 bp long and the last one 870 bp long. The second strategy cuts the alignment into 18 sub-datasets, with the first 17 sub-datasets 500 bp long and the last one 770 bp long. The third strategy splits the alignment into 23 sub-datasets, with the first 22 sub-datasets 400 bp long and the last one 470 bp long. The last strategy subdivides the alignment into 31 sub-datasets, with the first 30 sub-datasets 300 bp long and the last one 270 bp long. Phylogenetic analysis of the whole genome alignment and all of the sub-datasets was carried out using RAxML [26] under the GTRCAT approximation [27] and random starting trees. 1000, 600, 500, 400 and 300 rapid bootstrap replicates were performed for the full-length genome dataset, sub-datasets split using the first, second, third and fourth subdivision strategies, respectively. All other parameters were set to default. All of the trees are available on request from the authors. Trees were visualized using Dendroscope [28].

**Figure 1:**
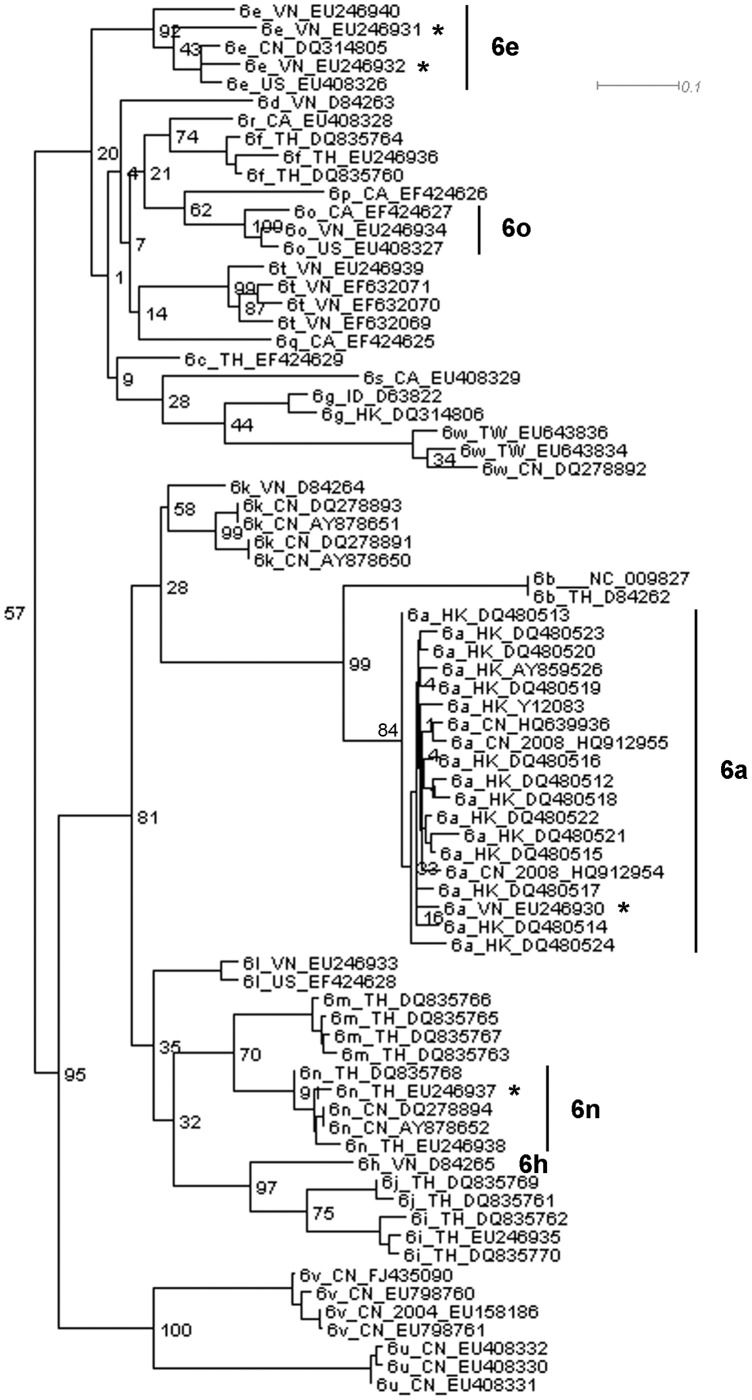
The genotype 6 part of the phylogenetic tree constructed using the first 600 bp of the alignment.

**Figure 2:**
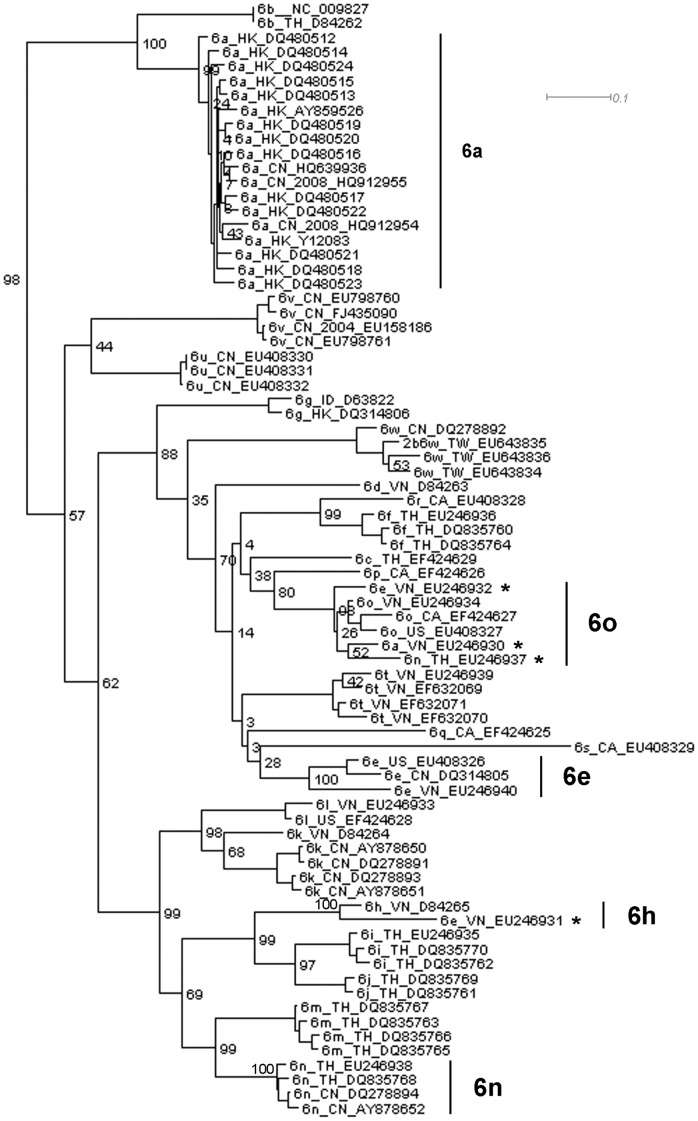
The genotype 6 part of the phylogenetic tree constructed using the last 827 bp of the alignment.

**Table 2:** Verification of the four novel inter-subtype recombinants by independent methods^a^.

GenBank No.	Recombinant	RDP	GENECONV	BootScan	Maxchi	Chimaera	SiSscan	3Seq
EU246930	6a/6o	***	***	***	***	***	***	***
EU246931	6e/6h	***	***	***	***	**	***	***
EU246932	6e/6o	***	***	***	**	*	**	**
EU246937	6n/6o	***	***	***	**	***	**	**

a ***means that the P value is smaller than 10^−20^ and **means that the P value is smaller than 10^−10^.

* means that the P value is smaller than 10^−5^.

**Figure 3:**
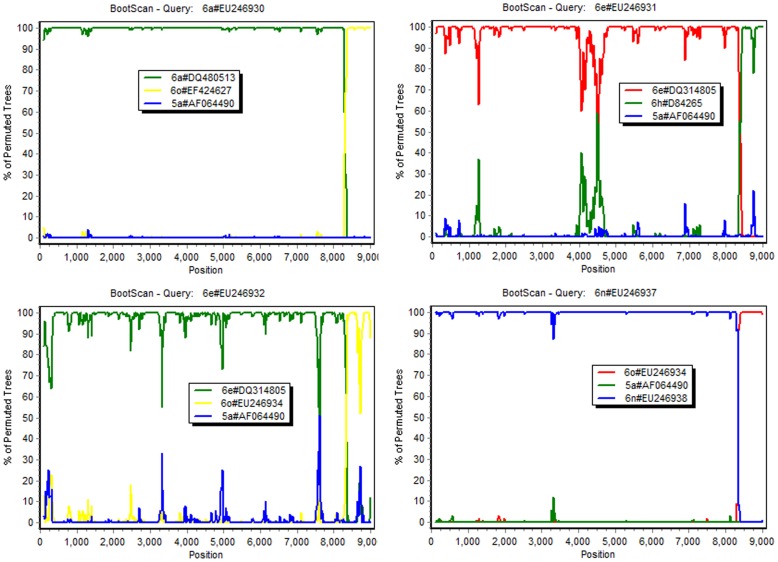
BootScan analysis of the novel recombinants and their possible parents. The coding region for the NS5B protein starts from position 7327 and ends at position 9099 in our alignment. Different colors represent sequences of different subtypes, two of which are the possible parental sequences and one of which (5a#AF06490) serves as an outgroup.

Information on these sequences, including genotype, subtype, and recombination, was downloaded from the database. This information was validated using the phylogenetic tree, constructed using the whole genome sequences, to correct potential genotype or subtype misclassifications and was used as background information. Subtyping information from each phylogenetic tree, constructed using the sub-datasets, was compared to the background information, on an individual basis. For each sequence, if all the information was concordant with the background information, this suggested the virus is not a recombinant. However, if a discrepancy between the background information and subtypes derived from the sub-datasets was identified, these sequences were analyzed further using multiple, independent computational methods described below.

**Table 3:** Breakpoints of the four novel inter-subtype recombinants^a^.

Recombinant	In alignment		Length (bp)	Sequence similarity (%)	
	Begin	End		Major parent	Minor parent
EU246930 (6a)	8345	9073	729	DQ480513(6a): 77.3	EF424627(6o): 93.8
EU246931 (6e)	8356	9019	664	DQ314805(6e): 75.9	D84265(6h): 94.1
EU246932 (6e)	8358	8977	620	DQ314805(6e): 83.5	EU246934(6o): 99.3
EU246937 (6n)	8372	9033	662	EU246938(6n): 79.4	EU246934(6o): 96.8

a Sequence similarity between the recombinants and their major and minor parents is estimated only using the recombined regions. The major and minor parents of the recombinants are identified by RDP.

**Table 4:** Likelihood mapping of NS5B gene sequences of genotype 6 HCV.

	HKY	TN	GTR
NS5B (7327–9099)	A_1_+A_2_+A_3_: 95.6%; A_7_: 1.8%	A_1_+A_2_+A_3_: 95.5%; A_7_: 1.8%	A_1_+A_2_+A_3_: 96.0%; A_7_: 1.2%
NS5B1 (7327–8357)	A_1_+A_2_+A_3_: 90.1%; A_7_: 5.3%	A_1_+A_2_+A_3_: 90.1%; A_7_: 5.4%	A_1_+A_2_+A_3_: 91.6%; A_7_: 3.5%
NS5B2 (8358–9099)	A_1_+A_2_+A_3_: 91.1%; A_7_: 4.9%	A_1_+A_2_+A_3_: 90.3%; A_7_: 5.6%	A_1_+A_2_+A_3_: 93.2%; A_7_: 2.7%

**Table 5:** Statistical tests for alternative topologies derived from the recombination events detected*.

Event	Recombinant	Trees derived from (nt)		Test 1 vs 2			Test 2 vs 1		
		Dataset1	Dataset2	-lk best	p-SH	c-ELW	-lk best	p-SH	c-ELW
EU246930	8345–9073	7327–8344	8245–9073	18837.90	<10^−4^	<10^−4^	12008.24	<10^−4^	<10^−4^
EU246931	8356–9019	7327–8355	8356–9019	20068.04	<10^−4^	<10^−4^	10942.59	<10^−4^	<10^−4^
EU246932	8358–8977	7327–8357	8358–8977	20495.72	<10^−4^	<10^−4^	10339.85	<10^−4^	<10^−4^
EU246937	8372–9033	7327–8371	8372–9033	19985.97	<10^−4^	<10^−4^	10887.61	<10^−4^	<10^−4^

* Only the NS5B gene regions were used for this analysis.

Because the putative novel recombinants belonged to genotype 6, only the sequences of genotype 6 HCV (n = 77) were used for verification. All the potential novel putative recombinants were verified in a single run using the program RDP 3 [29]. The methods used included RDP [30], GENECONV [31], BootScan [32], Maxchi [33], Chimaera [34], SiSscan [35] and 3Seq [36]. The breakpoints were also defined by RDP. Similarity between the recombinants and their possible major and minor parents was estimated using Bioedit. BootScan, embedded in Simplot [37], was used to visualize the relationships among the recombinants and their possible parents, with a sequence (AF064490) from genotype 5 serving as an outgroup.

To further verify these recombination events, we extracted the NS5B genes of genotype 6 HCV from the whole alignment and split the alignment into two sub-alignments according to the breakpoints identified: the non-recombinant region and the recombinant region. We constructed phylogenetic trees using the non-recombinant NS5B gene regions and the recombinant regions, respectively. This was performed using PhyML [38]. To test the alternative topologies derived, we performed the Kishino-Hasegawa (KH) test [39] and calculated expected likelihood weights [40] using Tree-Puzzle [41].

In addition, to exclude the possibility that the detected recombination events are caused by lack of phylogenetic signals in the 3′-end of genotype 6 HCV, we used the likelihood mapping method [42], implemented in Tree-Puzzle, to test whether the datasets used for detecting recombination events are suitable for phylogenetic analysis. Three models (HKY [43], TN [44] and GTR [45]) were used, respectively. Similarly, only the NS5B genes of genotype 6 HCV were used in this analysis.

## Results

### Phylogenetic Analysis of the Full-length Genome Sequences

Phylogenetic analysis of the 1278 full-length genome sequences supports the current classification of HCV into seven genotypes, 1–7 (File S1). The number of sequences belonging to genotype 1 was 993, accounting for approximately 78% of the whole dataset, while that of genotype 2 was 116 (9%). Genotypes 3–7 included 33, 47, 5, 77 and 1 sequence, respectively.

### Inter-genotype Recombination

By comparing phylogenetic signals from different subdivided fragments of the full-length genome sequences, we identified nine inter-genotype HCV recombinants. They belong to five recombination types, 2/5 (n = 2), 2b/6w (n = 1), 2b/1a (n = 1), 2b/1b (n = 1), and 2k/1b (n = 4), respectively (Table S2). All of these have been previously described [9]. No novel inter-genotype recombinants were found.

### Inter-subtype Recombination

Phylogenetic trees constructed using different sequence fragments can be used to find potential inter-subtype recombination events. In all, five inter-subtype recombinants were identified. The 1a/1c recombinant (AY651061) has already been reported [46] and was not further studied. The remaining four sequences, EU246930, EU246931, EU246932 and EU246937, are shown for the first time to be recombinants. These four sequences were isolated from Vietnam and Thailand and have been reported to belong to subtypes 6a, 6e, 6e and 6n, respectively [47]. Phylogenetic analysis of the full-length genome sequences confirmed this subtype classification (data not shown). However, phylogenetic trees estimated using the 600 bp (n = 15), 500 bp (n = 18), 400 bp (n = 23) and 300 bp (n = 31) fragments were consistent and demonstrated that EU246930, EU246931, EU246932 and EU246937 are 6a/6o, 6e/6h, 6e/6o, and 6n/6o recombinants, respectively (Table 1).

Figures 1 and 2 demonstrate how potential recombination events are identified from the trees. Figures 1 and 2 present the genotype 6 lineages of the phylogenetic trees constructed using the first fragment (600 bp in length) and the last fragment (827 bp in length) in the first sub-division strategy. In Figure 1, EU246930 (6a) is clustered within a lineage of 6a sequences and the bootstrap support value for this lineage is 84%. EU246931 and EU246932 (6e) fall within a cluster of 6e, with a bootstrap value of 92%, while EU246937 belongs to subtype 6n with a bootstrap value of 91%. However, in Figure 2, different phylogenetic relationships are found. EU246930 (6a), EU246932 (6e) and EU246937 (6n) are clustered with a lineage of subtype 6o sequences and the bootstrap support is 98%, while EU246931 (6e) forms a separate lineage with D84265 (6h) with 100% bootstrap support. Employing this approach, we analyzed all the trees and summarized the discordant phylogenetic signals suggesting evidence of recombination.

Further verification of these four recombinants was performed using RDP (Table 2). The four inter-subtype recombination events are supported by seven methods with significant p values (Table 2). The relationships within the recombinants with the potential major and minor parents identified by RDP were visualized using BootScan (Figure 3), which confirmed the recombination events.

Phylogenetic analyses and BootScan analysis indicate that the breakpoints of the four recombinants are located within the NS5B region (Table 1, Figure 3). Breakpoints of the four recombinants defined by RDP are consistent with the result obtained by phylogenetic analysis. However, the locations are not exactly the same in each case and the length of the recombined segments ranged from 620 bp for EU246932 to 729 bp for EU246930 (Table 3).

Results from the KH test and ELW were consistent and both of them supported that the phylogenies derived the non-recombinant region and the recombinant region were significantly different (Table 4).

Likelihood mapping analysis of NS5B gene sequences of genotype 6 HCV using different models were congruent. All of them showed that the tree-likeness of the NS5B gene was very high, with the sum of A_1_, A_2_, and A_3_ ranging from 95.5% to 96.0% (Table 5). In contrast, the value of A_7_, which is evidence to support the star-likeness, was relatively small, ranging from 1.2% to 1.8%. In particular, the recombinant regions (8358–9099) also displayed very high probability of tree-likeness (Table 5).

## Discussion

Recombination in HCV has been considered a rare event. This is supported by the observation of superinfection exclusion, where an established virus infection prevents or interferes with subsequent infection by a second virus [10]. The first naturally occurring inter-genotype HCV recombinant was identified in 2002 [16]. This recombinant became established and is still circulating in some European countries [21], [22]. So far, seven inter-genotype recombination types have been described [9]. Here, we identify nine inter-genotype recombinants and they belong to five inter-genotype recombination types, 2/5, 2b/6w, 2b/1a, 2b/1b and 2k/1b, respectively. All of these have been previously reported and no new or novel inter-genotype recombinants are found in this analysis.

So far, only one subtype of genotype 5, 5a, has been identified. The breakpoint of the 2/5 recombinants is identified to be at or near the NS2/NS3 junction, between residues 3420 and 3440 [48]. Our results confirm this finding. However, the sequence divergence between the 2/5 recombinants and 5a from position 3421 to the end of the genome is 34.5% (1%, standard deviation), which is higher than the 20% cutoff used to define a subtype. Therefore, it is likely that the 2/5 recombinants are derived from a putative subtype of genotype 5, rather than 5a. Further collecting and sequencing more HCV samples of genotype 5 is needed to reveal the real phylogenetic diversity of HCV and to trace the most likely parents of the 2/5 recombinants.

In our work, five inter-subtype recombinants were found through large-scale phylogenetic analyses. The 1a/1c recombinant sequence was identified in India and has already been reported [46]. However, the remaining four recombinants are described here for the first time. These recombination events were well supported by various recombination detection methods and were shown not to result from the lack of phylogenetic signal in the 3′-end of HCV genomes. Specifically, they represent four novel inter-subtype recombination types, 6a/6o, 6e/6o, 6e/6h and 6n/6o, respectively.

Although only a few HCV recombinants have been described, current evidence suggests that the NS2/NS3 junction may be a hotspot for HCV recombination [9]. However, a breakpoint has also been identified within NS5B [49] and this is mapped to position 8046 in our alignment which is different from the breakpoints identified in our study (8245, 8356, 8358 and 8372, respectively). Notably, while the breakpoints of the recombinants identified in our study are not identical, they are very close. At present, it is impossible to determine whether these recombinants have arisen from single or multiple recombination events.

Two previous studies have also shown that recombination can happen within a single subtype or a patient [50], [51]. Sentandreu et al. analyzed 17712 sequences from 136 serum samples derived from 111 patients and found approximately 11% of the samples were potential recombinant sequences [51]. On this basis, they concluded that recombination should be considered as a potentially important molecular mechanism for HCV to generate novel genetic variants. However, because our dataset has approximately 1300 sequences, it is extremely difficult to study detailed phylogenetic relationships for each sequence within a subtype using our approach and therefore we did not investigate intra-subtype recombination.

The subdivision of the whole HBV genome into numerous sub-datasets has previously termed “fragment typing” and has been used to identify putative HBV recombinants [52]. We have also recently used a similar approach to detect HBV recombination [7]. In this work, we applied four strategies to split the whole genome alignment into sub-datasets of different lengths, with different start and end points. The results obtained from the four strategies are broadly in agreement. Therefore, we consider our approach to be very robust for the detection of inter-genotype and inter-subtype HCV recombinants and this is particularly useful when large datasets with thousands of genome sequences are involved. However, this has two limitations. First, it may not be effective for the detection of small recombined fragments of less than 100 bp, because the shorter the alignment is, the lower the power and sensitivity of the phylogenetic analysis. Second, it is difficult to detect intra-subtype recombination using this method. For some subtypes, such as 1a and 1b where there are a few hundred sequences available, it is difficult to detect the incongruent phylogenetic signals by “eyeballing” the trees. In these cases, methods that are able to automatically detect potential recombination events, such as RDP, as used in this study, should be employed.

Previous computer simulation studies and empirical data have shown that different recombination detection methods have distinct features and no single method is best for all situations [34], [53]. In this work, seven methods used for verification of the results obtained from phylogenetic analyses. These methods are based on different rationales and have been classified into different classes [34], [53]. For example, RDP, BootScan and SiSscan are phylogeny-based, while GENECONV, Maxchi and Chimaera are substitution-based. Because all of these methods detected the four sequences as recombinants, this provides very convincing evidence that these recombinants have been properly designated.

In conclusion, we have performed a large scale phylogenetic analysis of 1278 full-length genome sequences to detect putative inter-genotype and inter-subtype recombinants. No new or novel inter-genotype recombinants were found. However, we have identified for the first time four novel inter-subtype recombinants. Our studies suggest that HCV recombination and its implications for both pathogenesis and clinical outcomes certainly warrant further study.

## Supporting Information

Table S1 **Four strategies to subdivide the alignment into sub-datasets.**
(XLSX)

Table S2 **List of the identified inter-genotype HCV recombinants.**
(DOCX)

File S1 **Phylogenetic analysis of 1278 complete HCV genome sequences.**
(TXT)
