# Supplementary material for: Recombination in Hepatitis C Virus: Identification of Four Novel Naturally Occurring Inter-Subtype Recombinants
Source: PLoS One. 2012 Jul 24;7(7):e41997. doi: 10.1371/journal.pone.0041997 (PMC3404033; doi:10.1371/journal.pone.0041997)
Supplement: Table S2 — List of the identified inter-genotype HCV recombinants. (DOCX) [file pone.0041997.s002.docx]

**TABLE S2** List of the identified inter-genotype HCV recombinants

| GenBank No. | Country/Region | Year | Recombination type | Reference |
| --- | --- | --- | --- | --- |
| AM408911 | France | - | 2/5 | ([6](#_ENREF_6)) |
| EF026073 | France | - | 2/5 |  |
| EU643835 | Tai Wan | - | 2b/6w | ([5](#_ENREF_5)) |
| JF779679 | United States | 2010 | 2b/1a | ([1](#_ENREF_1)) |
| AB622121 | Japan | 2010 | 2b/1b | ([7](#_ENREF_7)) |
| DQ364460 | Philippines | - | 2b/1b | ([3](#_ENREF_3)) |
| HQ537005 | Cyprus | 2005 | 2k/1b | ([2](#_ENREF_2)) |
| AY587845 | Russia | 1999 | 1b/2k | ([4](#_ENREF_4)) |
| HQ537006 | Cyprus | 2007 | 2k/1b | ([2](#_ENREF_2)) |

1. **Bhattacharya, D., M. A. Accola, I. H. Ansari, R. Striker, and W. M. Rehrauer.** 2011. Naturally occurring genotype 2b/1a hepatitis C virus in the United States. Virology journal **8:**458.

2. **Demetriou, V. L., E. Kyriakou, and L. G. Kostrikis.** 2011. Near-full genome characterisation of two natural intergenotypic 2k/1b recombinant Hepatitis C virus isolates. Adv Virol **2011:**710438.

3. **Kageyama, S., D. M. Agdamag, E. T. Alesna, P. S. Leano, A. M. Heredia, I. P. Abellanosa-Tac-An, L. D. Jereza, T. Tanimoto, J. Yamamura, and H. Ichimura.** 2006. A natural inter-genotypic (2b/1b) recombinant of hepatitis C virus in the Philippines. Journal of medical virology **78:**1423-1428.

4. **Kalinina, O., H. Norder, S. Mukomolov, and L. O. Magnius.** 2002. A natural intergenotypic recombinant of hepatitis C virus identified in St. Petersburg. Journal of virology **76:**4034-4043.

5. **Lee, Y. M., H. J. Lin, Y. J. Chen, C. M. Lee, S. F. Wang, K. Y. Chang, T. L. Chen, H. F. Liu, and Y. M. Chen.** 2010. Molecular epidemiology of HCV genotypes among injection drug users in Taiwan: Full-length sequences of two new subtype 6w strains and a recombinant form_2b6w. Journal of medical virology **82:**57-68.

6. **Legrand-Abravanel, F., J. Claudinon, F. Nicot, M. Dubois, S. Chapuy-Regaud, K. Sandres-Saune, C. Pasquier, and J. Izopet.** 2007. New natural intergenotypic (2/5) recombinant of hepatitis C virus. Journal of virology **81:**4357-4362.

7. **Yokoyama, K., M. Takahashi, T. Nishizawa, S. Nagashima, S. Jirintai, S. Yotsumoto, H. Okamoto, and M. Y. Momoi.** 2011. Identification and characterization of a natural inter-genotypic (2b/1b) recombinant hepatitis C virus in Japan. Archives of virology **156:**1591-1601.
